# The VGVAPG Peptide Regulates the Production of Nitric Oxide Synthases and Reactive Oxygen Species in Mouse Astrocyte Cells In Vitro

**DOI:** 10.1007/s11064-019-02746-z

**Published:** 2019-02-13

**Authors:** Konrad A. Szychowski, Jan Gmiński

**Affiliations:** 1 Department of Public Health, Dietetics and Lifestyle Disorders, Faculty of Medicine, University of Information Technology and Management in Rzeszow, Sucharskiego 2, 35-225 Rzeszow, Poland; 2 Department of Clinical Biochemistry and Laboratory Diagnostics, Institute of Medicine, University of Opole, Oleska 48, 45-052 Opole, Poland

**Keywords:** Elastin-derived peptides, VGVAPG, eNos, iNos, nNos, ROS

## Abstract

The products of elastin degradation, namely elastin-derived peptides (EDPs), are detectable in the cerebrospinal fluid of healthy individuals and in patients after ischemic stroke, and their number increases with age. Depending on their concentrations, both nitric oxide (NO) and reactive oxygen species (ROS) take part either in myocardial ischemia reperfusion injury or in neurovascular protection after ischemic stroke. The aim of our study was to determine the impact of VGVAPG peptide on ROS and NO production and expression of endothelial nitric oxide synthase (eNos), inducible nitric oxide synthase (iNos) and neuronal nitric oxide synthase (nNos) in mouse cortical astrocytes in vitro. Primary astrocytes were maintained in DMEM/F12 without phenol red supplemented with 10% fetal bovine serum. The cells were exposed to rising VGVAPG peptide concentrations, and ROS and NO production was measured. After 6 h (for mRNA) and 24 (for the protein) of exposure to 10 nM and 1 µM of the peptide, expression of nNos, iNos and eNos was measured. Moreover, the Glb1 siRNA gene knockdown method and Pioglitazone, a peroxisome proliferator-activated receptor gamma (Pparγ) agonist, were applied. Our study shows that the VGVAPG peptide decreased eNos, iNos and nNos mRNA and protein expression in mouse astrocytes in vitro. The VGVAPG peptide also decreased NO production while increasing ROS production in the cells. Furthermore, silencing of the Glb1 gene reversed all effects caused by the VGVAPG peptide. However, due to the lack of sufficient data explaining the molecular mechanism of action of the VGVAPG peptide in the nervous system, more studies in this area are necessary.

## Introduction

The degradation of elastin by elastolytic enzymes is a hallmark of tissue ageing and of cardiovascular diseases such as atherosclerosis, ischemic stroke and peripheral artery disease. It is also an important feature of the hereditary form of pulmonary emphysema and actinic dermatitis [1, 2]. The degradation products of elastin, i.e. elastin-derived peptides (EDPs), are detectable in the cerebrospinal fluid of both healthy subjects and patients with ischemic stroke [3, 4], which suggests their involvement in the nervous system’s ageing process. Locally produced EDPs in a wide range of concentrations exhibit a diversity of biological effects, acting as regulators of cell migration, differentiation or proliferation [5, 6]. To date, it has been well described that the main effects of EDPs are mediated through interaction with the 67-kDa elastin-binding protein (EBP), identified as an enzymatically inactive spliced variant of β-galactosidase (S-Gal) [7, 8].

Patients with ischemic stroke or transient ischemic attack are at increased risk for future cardiovascular events. Diabetes and insulin resistance have been identified as risk factors for stroke and myocardial infarction [9]. Moreover, diabetes, similar as ischemic stroke, increase in level of EDPs in patients sera [10]. Kernan et al. [9] showed that risk of stroke or myocardial infarction was lower in group of patients who received Pioglitazone, which improves insulin sensitivity [9]. Such data suggest a common pathomechanism of these diseases.

It is well described that diabetes and ischemic stroke are a large source of reactive oxygen species (ROS) [11]. However, ROS are generated in the cell through multiple mechanisms in both pathological and physiological conditions. ROS are generated via action of NADPH oxidase a process that consumes large amounts of oxygen which is then converted into the highly reactive superoxide radical (O_2_^−^) and hydrogen peroxide (H_2_O_2_) [12]. Activation of the most abundant proinflammatory enzyme, myeloperoxidase (MPO), generates secondary oxidants and chloroamines, which together with proteases such as elastase and gelatinase are used by the organism to destroy a broad range of microorganisms [13, 14]. However, during autoimmune disease, neutrophils can release ROS and proteases extracellularly, thus causing damage to tissues, degradation of elastin, proteins, lipids and dysregulation of oxidative homeostasis [13, 14]. To date, ROS induced by a xanthine and xanthine oxidase (XO) system caused alterations to collagen and glycosaminoglycan (GAG) biosynthesis in cultured human dermal fibroblasts [15]. Moreover, ROS increased mRNA expression of elastin in human skin fibroblasts in vitro [16]. Simultaneously, EDPs have been reported to induce ROS production in monocytes and human fibroblasts, thus accelerating the ROS generation process [17, 18]. On the other hand, EDPs also increase the activities of antioxidant enzymes such as superoxide dismutase (SOD), catalase (CAT) or glutathione peroxidase (GSH-Px), with a simultaneous increase in lipid peroxidation in human fibroblasts [19].

Nitric oxide (NO) is a very reactive molecule and is the first gaseous neurotransmitter to have been discovered in the brain (for a review, see Vincent and Hope [20]). NO is synthesized from l-arginine by enzymes called NO synthases (NOSs). To date, three isoforms have been described, i.e. neuronal NOS (nNOS, or type 1), inducible NOS (iNOS, or type 2) and endothelial NOS (eNOS, or type 3) [21]. NO modulates neuron survival and differentiation [22]. Moreover, a number of studies have shown that NO regulates neurotransmitter release and different aspects of synaptic dynamics, such as differentiation of synaptic specialisations, microtubule dynamics, architecture of synaptic protein organisation and modulation of synaptic efficacy [22, 23]. Depending on their concentrations, both NO and ROS take part in myocardial ischemia reperfusion injury or neurovascular protection after ischemic stroke [24, 25]. Unfortunately, the dark side of NO and ROS is that NO is promptly scavenged by O_2_^−^ and forms the highly toxic and reactive peroxynitrite which is responsible for a further increase of ROS and oxidative damage [25].

The Val-Gly-Val-Ala-Pro-Gly (VGVAPG) peptide, repeated several times in the elastin molecule, is the best characterised ligand that binds to EBP with high affinity [26]. To date, it has been shown that κ-elastin and the VGVAPG peptide do not affect ROS production in human dermal fibroblasts [18] or increase ROS production in murine monocytes [27]. Conversely, the VGVAPG peptide reduced ROS production in the neutrophils in control patients and in patients with stable chronic obstructive pulmonary disease (COPD) [28].

It has been noted that the VGVAPG peptide increases phosphorylation of eNOS in primary first-trimester cytotrophoblasts [29]. κ-elastin increases *eNOS* mRNA expression in human umbilical artery endothelial cells (HUAEC) [30]. Moreover, VGVAPG increases NO production in a time-, concentration- and receptor-dependent manner in the human microvascular endothelial cell-1 line (HMEC) [31].

Because there is a lack of information regarding the mechanism of action of the VGVAPG peptide in the nervous system, the aim of this study was to determine the VGVAPG peptide’s impact on ROS and NO production and on expression of eNos, iNos and nNos in mouse cortical astrocytes in vitro.

## Materials and Methods

### Reagents

Dulbecco’s Modified Eagle’s Medium/Ham’s Nutrient Mixture F12 (DMEM/F12) 1:1 (16-405-CVR) without phenol red was purchased from Corning (Manassas, USA). Trypsin, streptomycin, penicillin, amphotericin B, glycerol, 3-[(3-Cholamidopropyl)dimethylammonio]-1-propanesulfonate hydrate (CHAPS), 4-(2-Hydroxyethyl)piperazine-1-ethanesulfonic acid, *N*-(2-hydroxyethyl)piperazine-N′-(2-ethanesulfonic acid) (HEPES), dithiothreitol (DTT), NaCl, ethylenediaminetetraacetic acid (EDTA), dimethyl sulfoxide (DMSO), 2′,7′-dichlorodihydrofluorescein diacetate (H_2_DCFDA), asymmetric dimethylarginine (ADMA), NG-methyl-l-arginine (l-NMA), *N*-acetyl-l-cysteine (NAC) and Pioglitazone were purchased from Sigma-Aldrich (St. Louis, MO, USA). 4-Amino-5-methylamino-2′,7′-difluorofluorescein diacetate (DAF-FM DA) was purchased from Cayman Chemical (Michigan, USA). The FastStart Universal Probe Master (Rox) was purchased from ROCHE Applied Science (Mannheim, Germany). *Glb1* gene siRNA (sc-61342) was purchased from Santa Cruz Biotechnology (Santa Cruz, CA, USA). The VGVAPG and Val-Val-Gly-Pro-Gly-Ala (VVGPGA) peptides were synthesised by LipoPharm.pl (Gdańsk, Poland). Charcoal/dextran-treated fetal bovine serum (FBS) was purchased from EURx (Gdańsk, Poland). The High Capacity cDNA Reverse Transcription Kit and TaqMan® probes corresponding to specific genes encoding *Actb* (Mm00607939_s1), *nNos* (Mm01208059_m1), *iNos* (Mm00440502_m1) and *eNos* (Mm00435217_m1) were obtained from Life Technologies Applied Biosystems (Foster City, CA, USA). nNos (E-EL-M1281), iNos (E-EL-M0696) and eNos (E-EL-M0456) ELISA assays were obtained from Elabscience Biotechnology (WuHan, China). Stock solutions of the VGVAPG and VVGPGA peptides were prepared in DMSO and were added to DMEM/F12 medium. The final concentration of DMSO in the culture medium was always 0.1%.

### Astrocyte Cell Culture

The experiments were performed on mouse astrocyte cell culture isolated from fetuses (17/18 embryonal day) of pregnant female Swiss mice. The animals were anaesthetised with CO_2_ vapour and killed by cervical dislocation. After the isolation and digestion process, the cells were centrifuged and the pellet was suspended in DMEM/F12 1:1 without phenol red supplemented with 10% FBS, 100 U/mL penicillin, 0.10 mg/mL streptomycin and 250 ng/mL amphotericin B. Isolation was performed according to a previously described method which allows to obtain an almost pure culture of astrocytes (> 98% astrocytes) (Szychowski et al. 2018, supplementary data). The cells were seeded at a density of 20 × 10^6^ cells/75 cm^2^ in culture flasks. Cultures of the astrocyte cells were maintained at 37 °C in an atmosphere containing 5% CO_2_. In the logarithmic phase, after reaching 90% confluence, the cells were collected and frozen in liquid nitrogen. This procedure kills neurons in culture and leaves the astrocytes, which allows to collect a large number of cell banks and to store cells for further research. Before the experiment the cells were thawed and seeded in culture flasks and cultured for approximately 1 week to reach 80–90% confluence. Then the cells were trypsinised with 0.25% trypsin/0.05% EDTA and passaged on to an experimental plate.

### siRNA Gene Silencing Procedure

*Glb1* siRNA was used to inhibit *Glb1* gene expression in mouse primary astrocytes. siRNA was applied for 7 h at a final concentration of 50 nM in antibiotic-free medium containing the siRNA transfection reagent INTERFERin, according to a previously described method [32]. Cells with scrambled siRNA were used as the control. The effectiveness of *Glb1* mRNA silencing with the use of 50 nM specific siRNA was verified by measuring the mRNA levels. Previously, knockdown of the *Glb1* gene was estimated at 39% of the control mRNA and 60.05% of the control protein as described in [33, 34]. In present manuscript knockdown of the *Glb1* gene was estimated at 37.45% of the control mRNA (data not shown).

### Measurement of Nitric Oxide (NO) and Reactive Oxygen Species (ROS) Production

To determine the VGVAPG or VVGPGA (random sequence according Blaise et al. [35]) peptide’s ability to induce either NO or ROS production in the cells, 5 µM of DAF-FMDA (for NO measurement) or 5 µM of H_2_DCFDA (for ROS measurement) was applied. The cells were incubated with DAF-FMDA or H_2_DCFDA in serum-free and phenol red-free medium for 45 min before VGVAPG or VVGPGA peptide treatment. After 6 and 24 h of incubating the cells with the VGVAPG or VVGPGA peptide (5% CO_2_ at 37 °C), the culture medium was replaced with fresh medium to remove extracellular residual DAF-FM or DCF in order to reduce the fluorescence background. DAF-FM fluorescence was measured using a microplate reader (FilterMax F5) at excitation and emission maxima of 495 nm and 515 nm, respectively. DCF fluorescence was measured using a microplate reader (FilterMax F5) at maximum excitation and emission spectra of 485 nm and 535 nm, respectively. Similar experiments with inhibitors of the nitric oxide synthases ADMA and l-NMA have been made.

### Real-Time PCR Analysis of mRNAs Specific to Genes Encoding *eNos, iNos* and *nNos*

For the real-time PCR assay, astrocytes were seeded on 6-well plates and initially cultured for 24 h. After 6 h of exposure to 10 nM and 1 µM of VGVAPG, samples of total RNA were extracted from the astrocytes according to the manufacturer’s protocol and based on a previously described method [32]. After siRNA transfection, the procedure was performed after 6 h of exposure for a concentration of 10 nM and 1 µM of VGVAPG. Both RNA quality and quantity were determined spectrophotometrically at 260 nm and 280 nm (ND/1000 UV/Vis; Thermo Fisher NanoDrop, USA). Two-step real-time RT-PCR was conducted using the CFX Real Time System (BioRad, USA). The reverse transcription (RT) reaction was performed at a final volume of 20 µL with 180 ng of RNA (as a cDNA template) using the cDNA reverse transcription kit according to the manufacturer’s protocol. Products from the RT reaction were amplified using the FastStart Universal Probe Master (Rox) kit with TaqMan probes as primers for specific genes encoding *Actb, nNos, iNos* and *eNos* according to the manufacturer’s protocol. Amplification was carried out in a total volume of 20 µL containing 1.5 µL of the RT product; *Actb* was used as the reference gene. A standard qPCR procedure was utilized: 2 min at 50 °C and 10 min at 95 °C followed by 40 cycles of 15 s at 95 °C and 1 min at 60 °C. Random peptide sequence VVGPGA was used as control and did not affect *eNos, iNos* and *nNos* mRNA expression (data not shown).

### Enzyme-Linked Immunosorbent Assays (ELISA) for eNos, iNos and nNos

Levels of eNos, iNos and nNos proteins were determined after 24 h of exposure to 10 nM and 1 µM of VGVAPG or with co-treatment with Pioglitazone via enzyme-linked immunosorbent assays (ELISA). Specific detections of these proteins were obtained using ELISA, then the proteins were subjected to quantitative sandwich enzyme immunoassay. Assay was performed according to the manufacturer’s instructions (Elabscience Biotechnology; WuHan, China). Briefly, a 96-well plate was pre-coated with monoclonal antibodies specific to eNos, iNos or nNos. Standards and collected cell extracts were added to the wells and incubated for 90 min at 37 °C. Then, after removing the liquid, 100 µL of biotinylated detection antibodies were added for 60 min. After washing three times to remove any unbound substances, horseradish peroxidase-conjugated avidin was added. Following additional washing, 90 µL of the substrate solution was added to the wells for 15 min. Then 50 µL of the stop solution was added and absorbance was measured at 450 nm using a microplate reader (FilterMax F5). This value was proportional to the amount of eNos, iNos or nNos. The protein concentration was measured in each sample and was determined in triplicate for each sample using the Thermo Fisher NanoDrop device. Random peptide sequence VVGPGA was used as control and did not affect eNos, iNos and nNos protein expression (data not shown).

### Statistical Analysis

The data are presented as means ± standard deviation (SD) of three independent experiments. The treatment was repeated six times (n = 6) in each experiment. Data were presented as a percentage of the control. The data were analysed via one-way analysis of variance (ANOVA) followed by Tukey’s multiple comparison procedure *p*-probability value, ***p < 0.001, **p < 0.01 and *p < 0.05.

## Results

### Reactive Oxygen Species (ROS) and Nitric Oxide (NO) Production

**Fig. 1:**
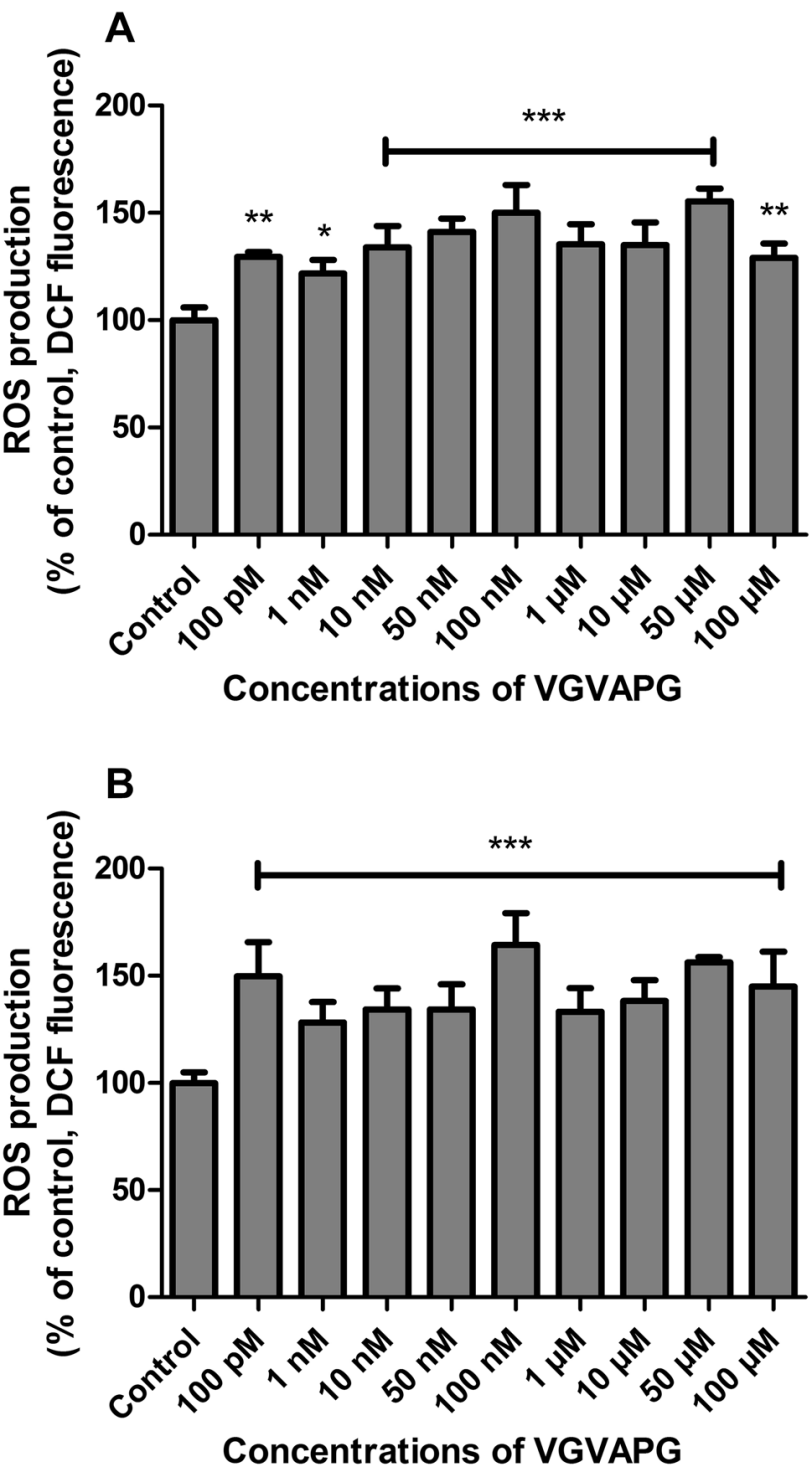
Effect of different (100 pM to 100 µM) VGVAPG peptide concentrations on DCF fluorescence (ROS measurement) in primary astrocytes; **a** after 6 h and **b** after 24 of exposure. Each point represents the mean ± SD of three independent experiments, each of which consisted of six replicates per treatment group. *P < 0.05, **P < 0.01, ***P < 0.001, versus the control cells

We observed a significant increase in ROS production after 6 and 24 h of exposure of mouse primary astrocytes to the VGVAPG peptide in concentrations ranging from 100 pM to 100 µM. After 6 h, the increase in ROS production was in the range of 121.77 to 155.50%, and after 24 h it was in the range of 128.31 to 164.47% of the control level (Fig. 1).

**Fig. 2:**
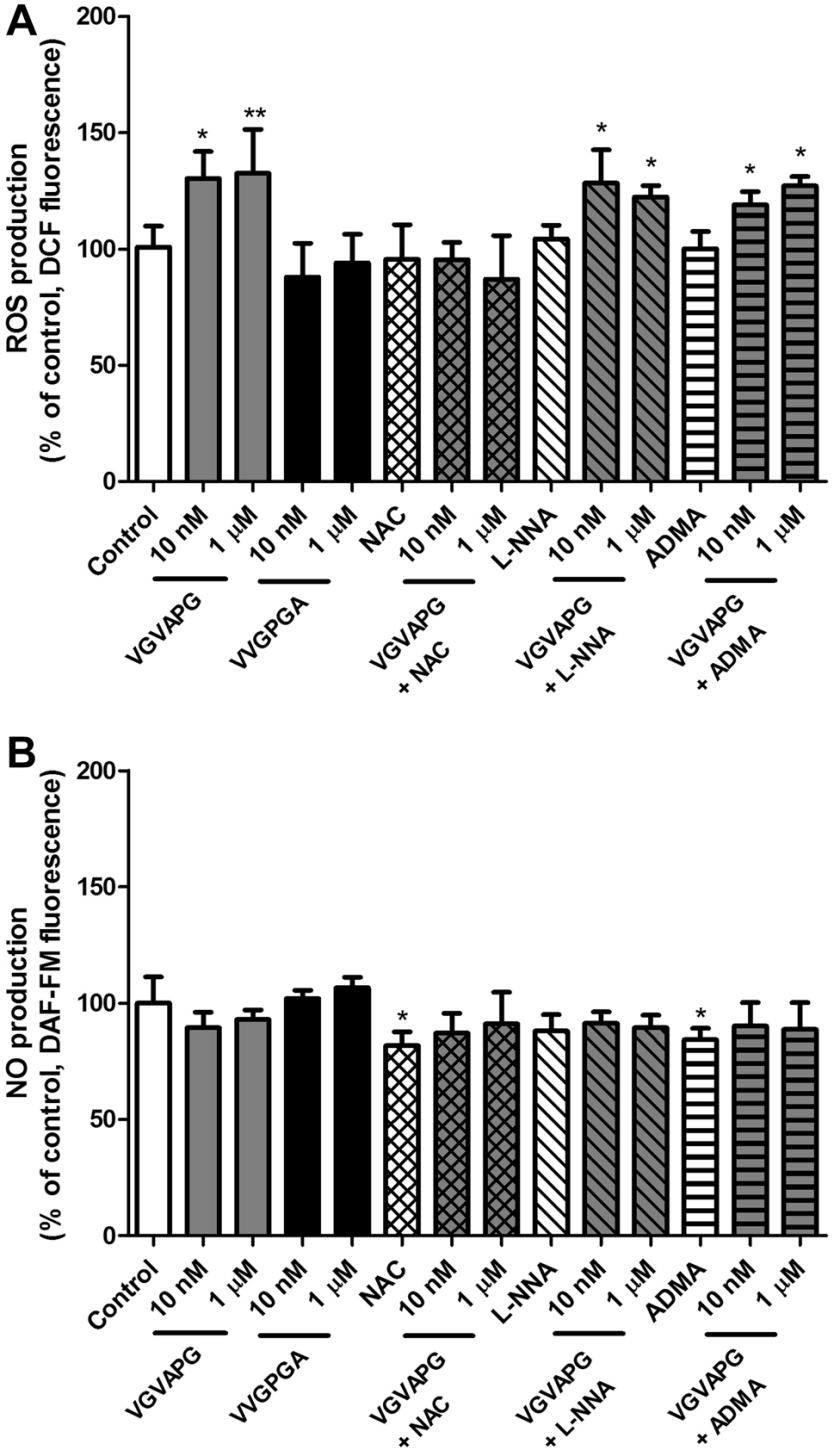
Effect of 10 nM and 1 µM of the VGVAPG peptide (grey bars), 10 nM and 1 µM of the random peptide sequence VVGPGA (black bars) and VGVAPG peptide co-treated with *N*-acetyl-l-cysteine (NAC) or NG-methyl-l-arginine (l-NMA) or asymmetric dimethylarginine (ADMA) on DCF fluorescence (ROS measurement) (**a**) or DAF-FM fluorescence (NO measurement) (**b**) in astrocytes after 6 h. Each point represents the mean ± SD of three independent experiments, each of which consisted of six replicates per treatment group. *P < 0.05; **P < 0.01; ***P < 0.001, versus the control cultures

After 6 h of the astrocytes’ exposure to 10 nM or 1 µM of the VGVAPG peptide there was an increase in ROS production by 26.55 and 27.40%, respectively, as compared to the control cells. Cells co-treated with *N*-Acetyl-l-cysteine (NAC) and 10 nM or 1 µM of the VGVAPG peptide prevented ROS formation (Fig. 2a). We observed no changes in NO production in cells exposed to 10 nM or 1 µM of VGVAPG or in those co-treated with NAC for 6 h (Fig. 2b).

After 3 and 6 h we observed no changes in ROS or NO production in cells exposed to random peptide sequence VVGPGA. Inhibitors of the nitric oxide synthases ADMA and L-NMA did not reduce ROS production stimulated by 10 nM or 1 µM of VGVAPG. Similar, 10 nM or 1 µM of VGVAPG do not affect NO production in cell co-treated with ADMA or l-NMA.

### siRNA Gene Silencing

#### ROS and NO Production

**Fig. 3:**
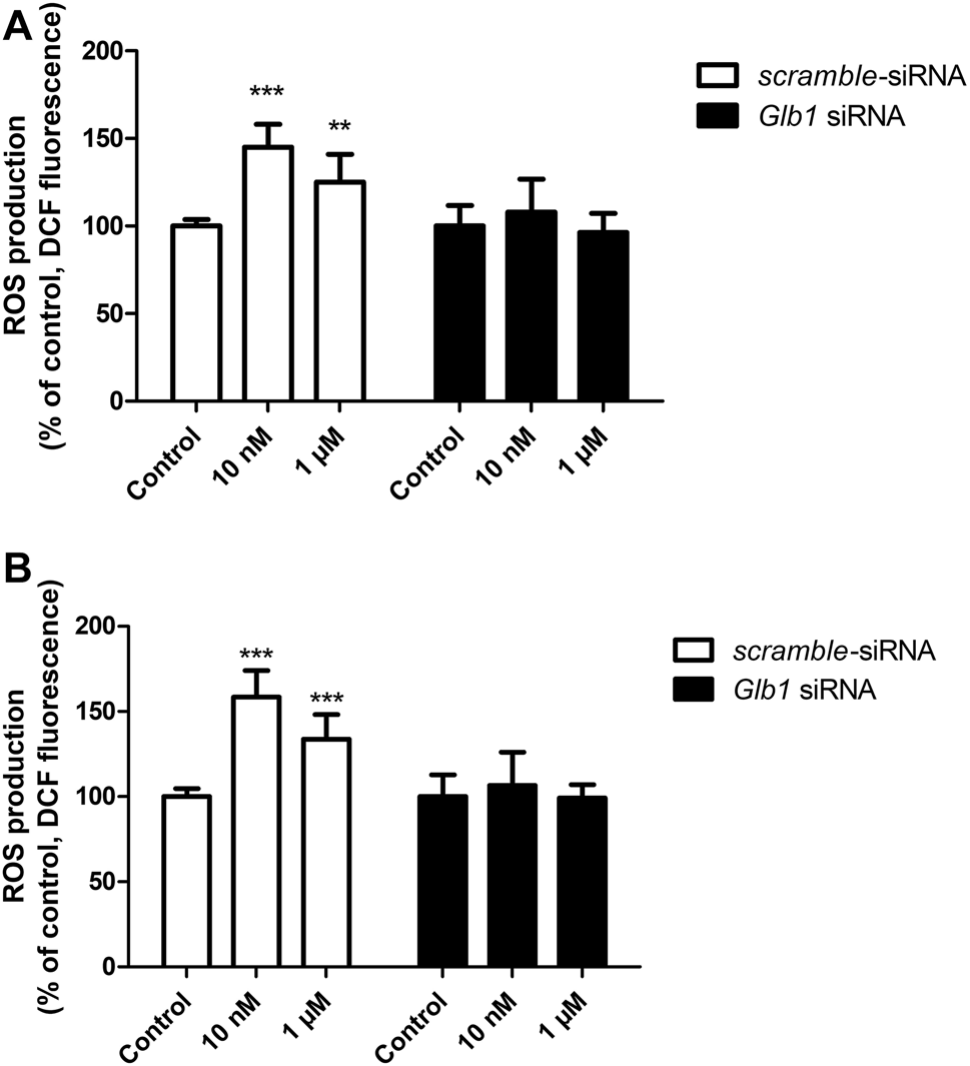
Effect of 10 nM and 1 µM of the VGVAPG peptide on DCF fluorescence (ROS measurement) in astrocytes; **a** after 6 h and **b** after 24. White bars represent cells transfected by scrambled siRNA, black bars represent cells transfected by *Glb1* siRNA. Each point represents the mean ± SD of three independent experiments, each of which consisted of six replicates per treatment group. *P < 0.05, **P < 0.01, versus the control cultures

After 6 h of exposure of mouse primary astrocytes to 10 nM and 1 µM of the VGVAPG peptide, we observed an increase in ROS production by 44.08 and 25.11%, respectively, as compared to the control. After 24 h ROS production potentate; therefore the increase in ROS was 58.41 and 33.81%, respectively, as compared to the control. After employing 50 nM of *Glb1* siRNA and cell stimulation with 10 nM and 1 µM of the VGVAPG peptide, we observed no changes in ROS production after 6 and 24 h of exposure to the peptide (Fig. 3).

**Fig. 4:**
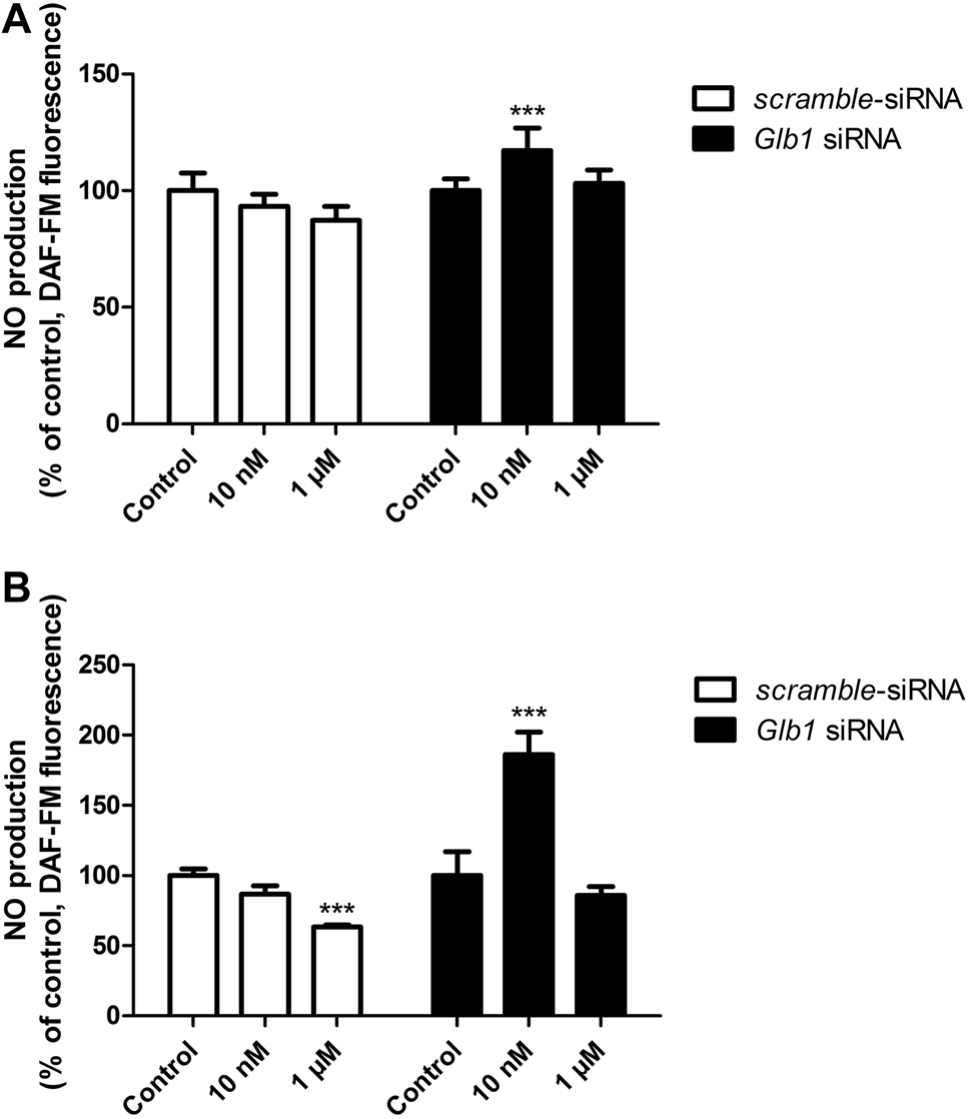
Effect of 10 nM and 1 µM of the VGVAPG peptide on DAF-FM fluorescence (NO measurement) in astrocytes; **a** after 6 h and **b** after 24. White bars represent cells transfected by scrambled siRNA, black bars represent cells transfected by *Glb1* siRNA. Each point represents the mean ± SD of three independent experiments, each of which consisted of six replicates per treatment group. ***P < 0.001, versus the control cultures

After 6 h of exposure of mouse primary astrocytes to 10 nM and 1 µM of the VGVAPG peptide, we observed no changes in NO production. However, after 24 h of exposure to 1 µM of VGVAPG, NO production decreased by 37.81% as compared to the control. After employing 50 nM of *Glb1* siRNA and cell stimulation with 10 nM of the VGVAPG peptide, we observed an increase in NO production after 6 and 24 h of exposure (an increase by 17.24 and 86.20%, respectively, as compared to the control) (Fig. 4).

#### *eNos, iNos* and *nNos* mRNA Expression

After 6 h of the mouse astrocytes’ exposure to 10 nM and 1 µM of the VGVAPG peptide, a decrease in *iNos* and *eNos* mRNA expression by 27.00 and 53.20%, respectively, was noted, whereas exposure to 1 µM of the VGVAPG peptide caused a decrease in *eNos* mRNA expression by 19.63%.

**Fig. 5:**
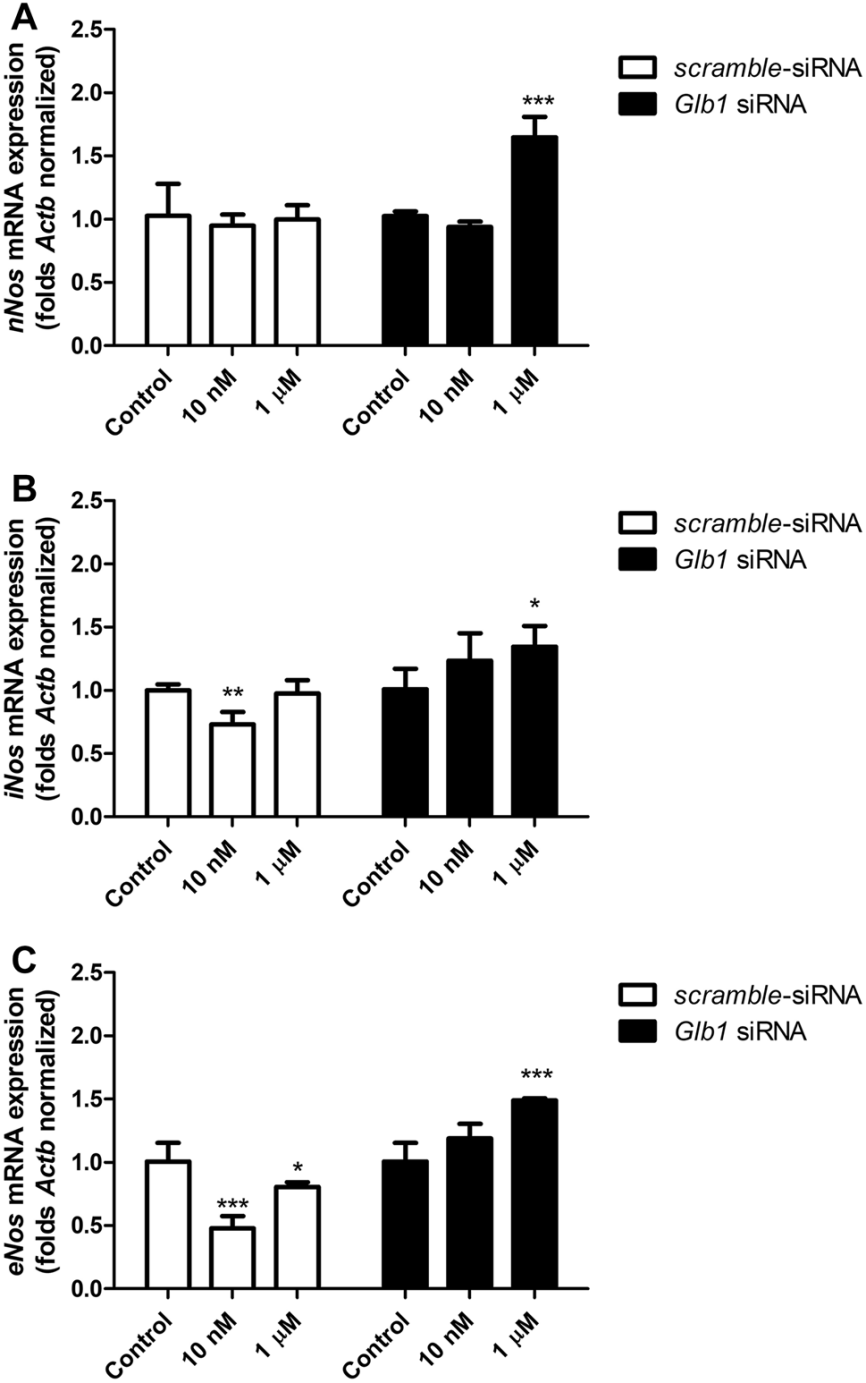
Effect of 10 nM and 1 µM of the VGVAPG peptide on mRNA expression of *nNos* (**a**), *iNos* (**b**) and *eNos* (**c**) after 6 h of exposure. White bars represent cells transfected by scrambled siRNA, black bars represent cells transfected by *Glb1* siRNA. mRNA expression was normalised to the *Actb* gene. Data are expressed as means ± SD of three independent experiments. *P < 0.05; **P < 0.01; ***P < 0.001 versus vehicle control

After employing 50 nM of *Glb1* siRNA and cell stimulation with 1 µM of the VGVAPG peptide, expression of *nNos, iNos* and *eNos* mRNA decreased by 64.70, 34.60 and 49.00%, respectively, as compared to the control (Fig. 5).

#### eNos, iNos and nNos Protein Expression

After 24 h of the mouse astrocytes’ exposure to 10 nM of the VGVAPG peptide, expression of nNos, iNos and eNos decreased by 0.55 ng/mL, 42.6 pg/mL and 27.16 pg/mL, respectively, as compared to the control.

**Fig. 6:**
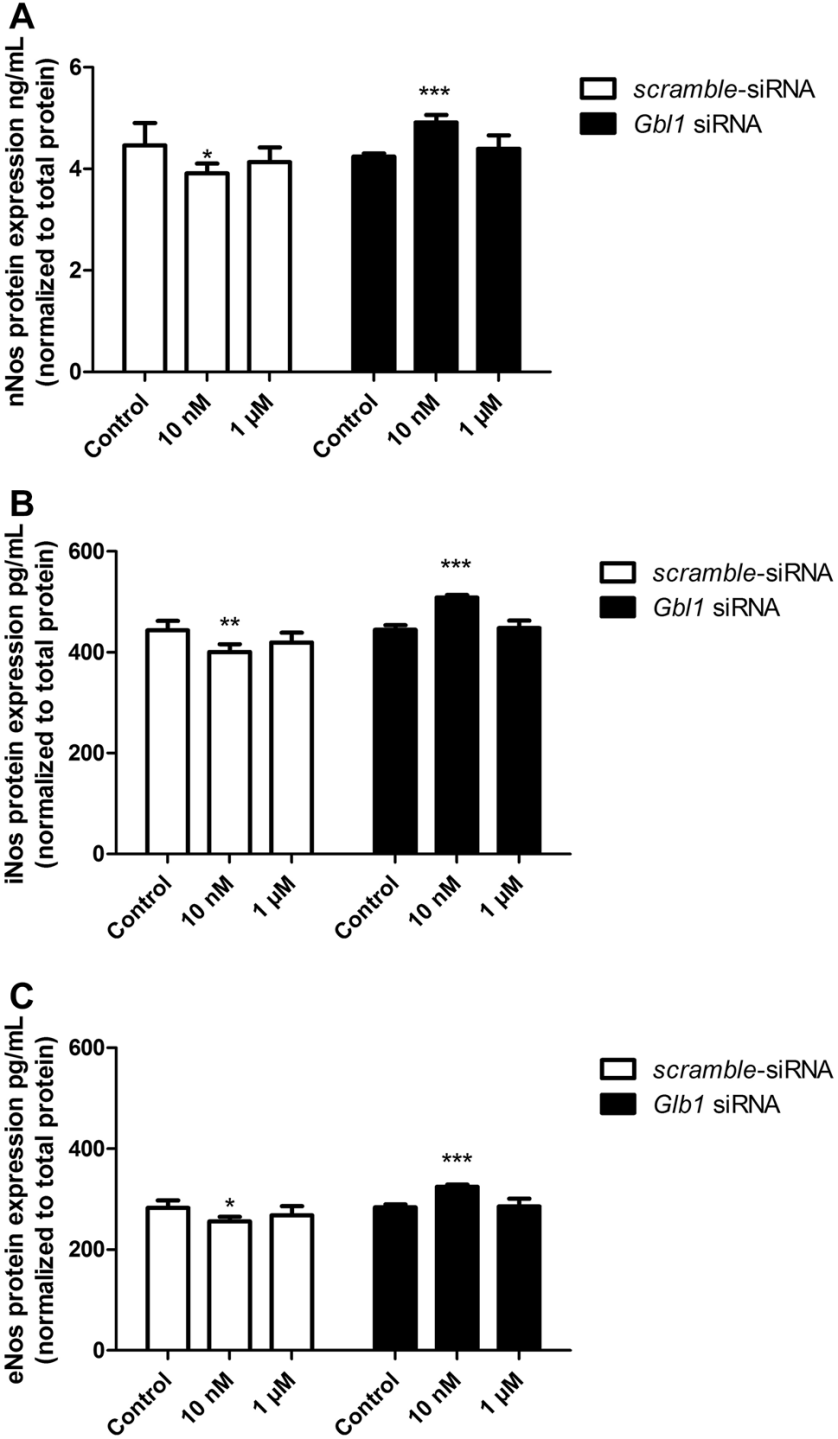
Effect of 10 nM and 1 µM of the VGVAPG peptide on protein expression of nNos (**a**), iNos (**b**) and eNos (**c**) after 24 h of exposure. White bars represent cells transfected by scrambled siRNA, black bars represent cells transfected by *Glb1* siRNA. Protein expression was normalised to the total protein level. Data are expressed as means ± SD of three independent experiments. *P < 0.05; **P < 0.01; ***P < 0.001 versus vehicle control

After employing 50 nM of *Glb1* siRNA and cell stimulation with 10 nM of the VGVAPG peptide, expression of nNos, iNos and eNos proteins increased by 0.68 ng/mL, 64.37 pg/mL and 40.85 pg/mL, respectively, as compared to the control (Fig. 6).

### eNos, iNos and nNos Protein Expression After Pioglitazone Stimulation

**Fig. 7:**
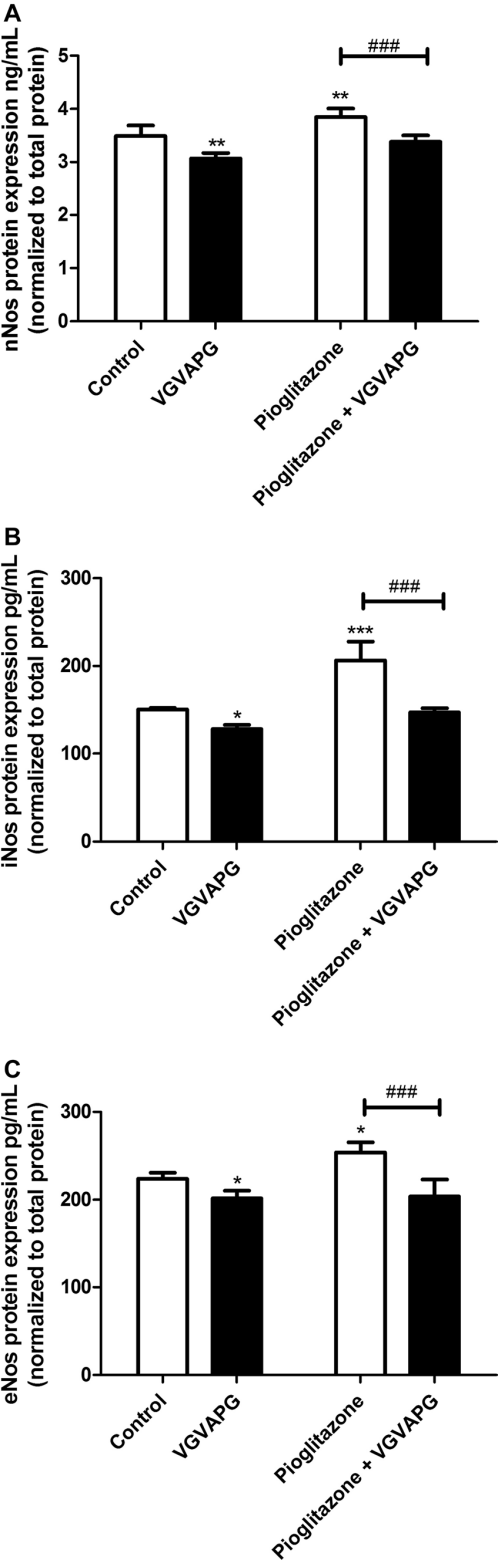
Effect of 10 nM of the VGVAPG peptide or co-treatment with 10 µM of Pioglitazone on protein expression of nNos (**a**), iNos (**b**) and eNos (**c**) after 24 h of exposure. Protein expression was normalised to the total protein level. Data are expressed as means ± SD of three independent experiments. *P < 0.05; **P < 0.01; ***P < 0.001 versus vehicle control, ###P < 0.001 versus Pioglitazone-treated cells

After 24 h of exposing the mouse astrocytes to 10 nM of the VGVAPG peptide, expression of nNos, iNos and eNos decreased by 0.42 ng/mL, 22.03 pg/mL and 22.04 pg/mL, respectively, as compared to the control. Our experiments showed that 10 µM of Pioglitazone increased nNos, iNos and eNos protein expression (0.36 ng/mL, 56.21 pg/mL and 29.91 pg/mL, respectively) as compared to the control. After cell co-treatment with 10 nM of VGVAPG and 10 µM of Pioglitazone, expression of nNos, iNos and eNos proteins did not change as compared to the control (Fig. 7).

## Discussion

Our experiments are the first to show that the VGVAPG peptide in a wide range of concentrations (100 pM to 100 µM) increased ROS production in astrocytes. To date, it has been described that EDPs induce ROS production in murine monocyte and human fibroblasts [17, 18, 27]. Gmiński et al. [19] stated that κ-elastin increased both the activity of antioxidant enzymes (SOD, CAT, GSH-Px) and the lipid peroxide concentration within fibroblasts [19]. On the other hand, EDPs decrease ROS production in human neutrophil cells, which suggests that EDP effects are cell-/tissue-dependent [28]. An increased level of antioxidant enzymes can be an effect of EDP-elevated ROS production in cells and can be responsible for a reduction in ROS production over a long time frame.

Based on the literature data and on our previous research results, we decided to use 10 nM and 1 µM of the VGVAPG peptide, which is close to concentrations recorded in tissues in physiological and pathological conditions [3, 33]. Our research shows that *N*-acetyl-l-cysteine (NAC), which has strong free radical scavenging properties, reduced ROS production caused by the VGVAPG peptide in astrocytes. Asymmetric dimethylarginine (ADMA) and NG-methyl-l-arginine (l-NMA) (inhibitors of NO productions) do not affect ROS production stimulated by VGVAPG peptide. Our data suggest that ROS production is independent from the activity of nitric oxide synthases. Interestingly, we observed that the VGVAPG peptide slightly decreased NO production. To date, it has been well documented that increased ROS production can accelerate NO degradation due to decreased expression of eNOS or its inactivation by dephosphorylation of Ser1177 [36]. The reaction of NO with O_2_^−^ is approximately three times faster than between O_2_^−^ and the SOD enzyme [37]. NO is promptly scavenged by ROS to form the highly toxic and reactive peroxynitrite, which is responsible for a further increase in ROS [25]. This phenomenon can explain the data we obtained here, where over time the amount of ROS increases while the amount of NO decreases. Several authors described a decrease in the NO level through eNOS uncoupling by ROS [25, 38]. Uncoupled eNOS is a phenomenon that is characterised by diversion of electron transfer within the eNOS molecule from l-arginine oxidation, which results in a reduction of molecular oxygen to form superoxide instead of NO [39]. Under stroke, peroxynitrite formation may cause damage to the mitochondria, vascular endothelium and smooth muscle cells [40]. However, activation of the apoptotic process in astrocytes was not detected in our previous study [33]. To date, most publications concerning the EDP mechanism of action have described an increase in NO production. Robinet et al. [5] stated that κ-elastin and the VGVAPG peptide increased NO production in rat cardiomyocytes (RCs) and human coronary artery endothelial cells (HCAECs) [5]. Similarly, VGVAPG and tropoelastin also increased NO production in HMEC and bovine aortic endothelial cells (BAECs), respectively [31, 41]. However, in the brain in the early stages of haemorrhage and ischaemic stroke, a global decrease in the NO level occurs and corresponds to an increase in P-selectin levels, which promotes platelet aggregation and fibrin deposition and leads to microthrombosis formation [42]. This supports our suggestion that the VGVAPG peptide initiates the healing process in astrocytes [33]. Furthermore, it is well described that a decreased level of NO in the brain is crucial for activating different survival pathways, e.g. soluble guanylate cyclase (sGC), protein kinase G (PKG), phosphatidylinositol-3-kinase/serine/threonine kinase (PI3K/AKT) and mitogen-activated protein kinase/extracellular signal-regulated kinase (MEK/ERK) in cells [23].

It is known that EBP (an alternatively spliced form of the *Glb1* gene) is the main molecular receptor for the VGVAPG peptide, therefore we decided to use the *Glb1* siRNA silencing procedure. The application of *Glb1* siRNA reduced ROS generated by the VGVAPG peptide to the control level, moreover, gene knockout increased NO production in the astrocytes. Due to the crucial role of NO in stroke and the brain metabolism, we decided to study mRNA and protein expression of nNos, iNos and eNOS in astrocytes. Our experiments are the first to show that 10 nM of the VGVAPG peptide decreased *iNos* and *eNos* mRNA expression. However, employing *Glb1* siRNA prevented a decrease in *iNos* and *eNos* mRNA expression. Moreover, *Glb1* gene silencing enhanced *nNos, iNos* and *eNos* mRNA expression in cells stimulated by 1 µM of the VGVAPG peptide. Similarly, on the protein level, 10 nM and 1 µM of VGVAPG decreased nNos, iNos and eNos protein expression, and silencing of the *Glb1* gene reversed this process. Our results confirm the involvement of EBP in the VGVAPG peptide mechanism of action and suggest the involvement of another cellular receptor in signal transduction. This is probably galectin-3, which is a second receptor for the VGVAPG peptide [43]. Toupance et al. [43] also described the galectin-3 receptor as being involved in lung cancer cell chemotaxis and migration [43]. Activation of this receptor could be important in the migration of astrocytes and in the formation of a glial scar. Data pertaining to nitric oxide synthase expression after EDP stimulation are very limited. To date, it has been noted that in patients with bicuspid aortic valve disease, expression of the eNOS protein in aortic endothelial cells significantly decreased [44]. However, as in the case of the NO level, the papers mainly described an increase in NO synthase expression. Garczorz et al. [30] showed that κ-elastin increased eNOS mRNA expression but did not change protein expression in HUAEC cells [30]. Similarly, Desforges et al. [29] showed that 1 µg/mL of VGVAPG increased *eNOS* expression in human trophoblast cells [29].

Our data show that astrocytes treated with Pioglitazone increase protein expression of nNos, iNos and eNos synthases. The effect of Pioglitazone was reversed by 10 nM of the VGVAPG peptide. Our previous research showed that 50 nM of the VGVAPG peptide decreased peroxisome proliferator-activated receptor gamma (Pparγ) expression caused by Pioglitazone and Rosiglitazone [34]. Such data suggest that VGVAPG activates molecular pathways that may then compete with other pathways activated by PPARγ agonists. To date, it has been described that natural and synthetic PPARγ agonists such as 15-Deoxy-Δ-12,14-prostaglandin J2 (15d-PGJ2) or TZD (Rosiglitazone, Ciglitazone and Pioglitazone) dependently affect NO production in microglial and astrocyte cells [45]. Storer et al. [45] also stated that Pioglitazone in a range of 125 to 200 µM decreased NO production stimulated by lipopolysaccharide (LPS) in astrocytes. Interestingly, 50 µM of Pioglitazone and 100–150 µM of Rosiglitazone increased NO production stimulated by LPS. Similarly to our results, Goya et al. [46] showed that 10 µM of Pioglitazone slightly increased protein expression of eNOS in HUVECs [46]. Moreover, Troglitazone in a 5 µM concentration increased eNOS protein expression more than at a concentration of 10 µM. Therefore, due to the hormesis phenomenon, we can assume that the VGVAPG peptide and Pioglitazone may act more strongly and/or in a different manner at low concentrations. We used 10 µM of Pioglitazone in our experiment, as we believe that these are normal results for low concentrations of Pioglitazone. To date, in high micromolar concentrations, TZDs inhibit the expression of various inflammatory proteins, e.g. interleukin-1β (IL-1β), interleukin (IL-6) and tumour necrosis factor alpha (TNFα) in astrocytes [45, 47]. They also prevent the induction of pro-inflammatory transcription factors such as the nuclear factor kappa-light-chain-enhancer of activated B cells (NF-κB), activator protein-1(AP-1) and signal transducer and activator of the transcription (STAT) protein in other cell types [48]. Interestingly, EDP from the bulbus arteriosus of bonitos partially suppressed hypertensive vascular injuries, although it did not delay the occurrence of severe hypertension in stroke-prone spontaneously hypertensive rats [49]. Such data support our previous hypothesis that probably the VGVAPG peptide’s presence in the brain activates astrocyte pro-survival/healing pathways [33].

## Conclusions

Our study shows, for the first time, that the VGVAPG peptide decreases eNos, iNos and nNos mRNA and protein expression in mouse astrocytes in vitro. The VGVAPG peptide also decreases NO production and increases ROS production in cells. Furthermore, silencing of the *Glb1* gene reverses all effects caused by the VGVAPG peptide, which suggests the involvement of another receptor and its mechanism of action. Cells treated with Pioglitazone increased NO synthase protein expression which had been reversed by VGVAPG. Our paper provides more evidence to support the hypothesis that the VGVAPG peptide is involved in activating astrocyte pro-survival/healing pathways. However, due to the lack of sufficient data explaining the molecular mechanism of action of the VGVAPG peptide in the nervous system, more studies regarding this topic are necessary.

## Abbreviations

CAT: Catalase
CNS: Central nervous system
CSF: Cerebrospinal fluid
DMSO: Dimethyl sulfoxide
EBP: Elastin-binding protein
EDPs: Elastin-derived peptides
eNos: Endothelial nitric oxide synthase
FBS: Fetal bovine serum
*Glb1*: Gene for beta-galactosidase
GSH-Px: Glutathione peroxidase
iNos: Inducible nitric oxide synthase
MMP: Matrix metalloprotease
nNos: Neuronal nitric oxide synthase
NO: Nitric oxide
PBS: Phosphate-buffered saline
PPARγ: Peroxisome proliferator-activated receptor gamma
ROS: Reactive oxygen species
siRNA: Small interfering RNA
SOD: Superoxide dismutase
TIMP: Tissue inhibitor of metalloproteinase
VGVAPG: Val-Gly-Val-Ala-Pro-Gly
β-Gal: Beta-galactosidase
